# Loganin Ameliorates Cholestatic Liver Fibrosis by Inhibiting Hepatic Stellate Cell Autophagy and Activation Through EGFR Signaling

**DOI:** 10.1155/mi/7967581

**Published:** 2026-08-17

**Authors:** Xinwen Li, Huiling Zhang, Chao Zhang, Xiaomin Zhang, Jiefeng He

**Affiliations:** ^1^ Department of Pharmacy, Shanxi Bethune Hospital, Shanxi Academy of Medical Sciences, Tongji Shanxi Hospital, Third Hospital of Shanxi Medical University, Taiyuan 030032, China; ^2^ Tongji Hospital, Tongji Medical College, Huazhong University of Science and Technology, Wuhan 430030, China, hust.edu.cn; ^3^ Department of Gastroenterology, Shanxi Provincial People’s Hospital, Taiyuan, China, spph-sx.com; ^4^ Third Hospital of Shanxi Medical University, Shanxi Bethune Hospital, Shanxi Academy of Medical Sciences, Tongji Shanxi Hospital, Taiyuan 030032, China; ^5^ Department of Hepatobiliary Surgery, Shanxi Bethune Hospital, Shanxi Academy of Medical Sciences, Tongji Shanxi Hospital, Third Hospital of Shanxi Medical University, Taiyuan 030032, China

**Keywords:** autophagy, EGFR, hepatic stellate cells, liver fibrosis, loganin

## Abstract

Liver cirrhosis represents the irreversible end‐stage of various chronic liver diseases, whereas hepatic fibrosis, characterized by hepatic stellate cell (HSC) activation, constitutes the critical pathological intermediate in this progression. In this study, we investigated the antifibrotic effects of loganin, a natural iridoid glycoside, and elucidated its underlying mechanisms in bile duct ligation (BDL)–induced cholestatic liver injury and hepatic fibrosis and in TGF‐β1–stimulated HSCs. Sprague–Dawley rats subjected to BDL surgery received loganin for 14 days, resulting in marked amelioration of hepatomegaly, inflammatory infiltration, hepatocyte necrosis, and collagen deposition, alongside restoration of serum alanine aminotransferase (ALT), aspartate aminotransferase (AST), and total bilirubin (T‐BIL) levels and suppression of fibrogenic gene expression (collagen type I alpha 1 chain [COL1α1], alpha‐smooth muscle actin [α‐SMA], and fibronectin). Moreover, loganin attenuated hepatic autophagy, as evidenced by reduced Beclin‐1 expression and LC3B‐II/I ratio, with decreased colocalization of α‐SMA and LC3B. Bioinformatics analysis and molecular docking indicated that epidermal growth factor receptor (EGFR) may be the target protein through which loganin exerts its antifibrotic effect. Experimental validation demonstrated that loganin suppressed BDL‐induced EGFR phosphorylation in vivo. In TGF‐β1–activated HSC‐T6 cells, loganin inhibited cell activation, downregulated fibrosis markers, impaired autophagic flux, and suppressed TGF‐β1–induced upregulation of Beclin‐1 and LC3B‐II/I. Critically, cotreatment with the EGFR agonist EGF reversed the autophagy‐inhibitory and antifibrotic effects of loganin, whereas the EGFR inhibitor AG‐1478 mimicked loganin’s actions, confirming EGFR signaling as the functional mediator. Overall, these results indicated that loganin could alleviate liver injury and hepatic fibrosis in BDL rats by inhibiting autophagy and activation of HSCs, and this protective effect is mediated by the EGFR pathway.

## 1. Introduction

Cirrhosis is an irreversible end‐stage consequence of various chronic liver diseases in advanced stages [1], and liver fibrosis is the pivotal pathological link underlying the progression of chronic liver disease to cirrhosis [2, 3]. The main cause of liver fibrosis is hepatocyte injury caused by various reasons, leading to degeneration, autophagy, activation, and proliferation of hepatic stellate cells (HSCs), excessive production of extracellular matrix, and visceral collagen deposition, which will lead to excessive formation of fibrous septa and nodules [4]. Hepatic metabolic and synthetic functions subsequently decline, resulting in an increase in bilirubin and a reduction in the synthesis of thrombopoietin, as well as sinus microcirculation obstruction and portal vein pressure elevation, eventually leading to hepatic failure [1]. Without effective intervention on liver fibrosis, the continuous progression of fibrous deposition will eventually impair hepatic metabolic and synthetic functions, leading to liver failure [1, 5]. Therefore, exploring the pathogenesis and early diagnosis of liver fibrosis, as well as inhibiting its progression, is of great significance for the prevention and treatment of cirrhosis.

Loganin (CAS: 18524‐94‐2), an iridoid glycoside derived from *Cornus officinalis* [3], has been reported to exert multiple pharmacological effects, including hepatoprotection, antidiabetes, and anti‐inflammation [6, 7]. Importantly, accumulating evidence indicates that loganin possesses antifibrotic potential: It can reduce Ang II–induced cardiac fibrosis and bleomycin‐induced pulmonary fibrosis [8, 9] and alleviate liver inflammation, fat accumulation, and liver tissue fibrosis in a mouse model of nonalcoholic steatohepatitis [10]. Additionally, loganin exerts a protective effect against hepatic oxidative stress in type 2 diabetes by regulating the expression of factors associated with oxidative stress, inflammation, and apoptosis [7]. However, whether loganin exerts therapeutic effects against cholestasis‐induced liver injury and hepatic fibrosis—specifically in the bile duct ligation (BDL) model—remains to be elucidated, representing the central objective of the present investigation.

The activation of HSCs constitutes the fundamental cellular events driving liver fibrogenesis [11]. Upon activation, HSCs acquire a contractile, secretory phenotype characterized by alpha‐smooth muscle actin [α‐SMA] expression and TGF‐β1 production, thereby establishing a self‐perpetuating autocrine loop that amplifies fibrotic signaling [12]. Recent advances have identified autophagy as a critical metabolic adaptation that fuels HSC activation during liver injury, promoting cell survival, lipid droplet degradation, and energy generation required for myofibroblast differentiation [13, 14]. Notably, impaired autophagic flux, characterized by lysosomal dysfunction and autophagosome accumulation, has been implicated in the pathogenesis of multiple fibrotic disorders. Given that loganin restores autophagic flux in chronic constriction injury‐induced neuropathy [15] and given its established inhibitory effects on TGF‐β1–induced renal tubulointerstitial fibrosis [16] and bleomycin‐induced pulmonary fibrosis [9], we hypothesized that loganin may similarly modulate the autophagy and activation of HSCs to ameliorate hepatic fibrosis.

In this study, we used a rat model of BDL‐induced hepatic fibrosis and TGF‐β1–stimulated HSC‐T6 cells to systematically investigate the antifibrotic effects and underlying mechanisms of loganin. Our results demonstrated that loganin markedly inhibits HSC activation and restores autophagic flux, with the epidermal growth factor receptor (EGFR) signaling pathway acting as a key mediator of its protective effects against liver injury and hepatic fibrosis.

## 2. Methods and Materials

### 2.1. Establishment of a Liver Fibrosis and Injury Rat Model

Male Sprague–Dawley rats (8 weeks old) were supplied standard rat feedstuff and water ad libitum and were randomly divided into the following groups: sham, sham + loganin, BDL, and BDL + loganin. After isoflurane anesthesia, the choledochal duct of the experimental animal was isolated and then severed by double ligation [4, 17]. According to the previous study [10], rats were intraperitoneally injected with 7 mg/kg loganin (purity ≥ 98%, Aladdin, Shanghai, China) daily for 14 days. The rats in the sham group only underwent abdomen‐open surgery without ligation and were given the same volume of solvent. At termination, all rats were sacrificed using carbon dioxide. All animal experimental procedures were approved by the Ethics Committee of Shanxi Bethune Hospital (Approval Number YXLL‐2022‐006).

### 2.2. Histological Assessment

The fixed liver tissue was embedded in paraffin and cut into sections. Liver sections were deparaffinized and stained with hematoxylin (Solarbio, Beijing, China) and eosin (Sangon Biotech, Shanghai, China) (H&E), Sirius Red, or Masson, respectively. Liver pathology was photographed with a BX53 microscope (200×) (Olympus, Tokyo, Japan).

### 2.3. Measurement of Hepatic Injury

The hydroxyproline (HYP) concentration of liver tissue was assessed by a HYP assay kit (Nanjing Jiancheng, Nanjing, China) according to the manufacturer’s instructions. The content of alanine aminotransferase (ALT), aspartate aminotransferase (AST), and total bilirubin (T‐BIL) in serum was determined using the corresponding kits purchased from Nanjing Jiancheng (Nanjing, China).

### 2.4. Network Pharmacology Analysis

The target proteins of loganin were predicted using the SwissTargetPrediction online website (http://swisstargetprediction.ch/). Liver fibrosis–related target proteins were predicted using GeneCards (http://geneanalytics.genecards.org/). The overlapping target proteins were visualized using the Venny online tool (https://www.xiantaozi.com/products). The protein–protein interaction (PPI) network analysis of target genes was performed and visualized using the STRING 12.0 online tool (https://string-db.org/) [18] with an interaction score ≥ 0.4. Subsequently, the Gene Ontology (GO) and Kyoto Encyclopedia of Genes and Genomes (KEGG) enrichment analyses were carried out using the Database for Annotation, Visualization, and Integrated Discovery (DAVID) knowledgebase (https://david.ncifcrf.gov/home.jsp) [19] to assess their functional catalogs. Enrichment analysis was visualized using the bioinformatics online tool (http://www.bioinformatics.com.cn/).

### 2.5. Cell Culture

Rat liver astrocyte HSC‐T6 cells were cultured in DMEM (Servicebio, Wuhan, China) containing 10% fetal bovine serum (FBS, Tianhang Biotechnology, Huzhou, China) in an incubator with 5% CO_2_ at 37°C. Cells in the TGF‐β1 group were treated with 10 ng/mL TGF‐β1 (Sino Biological Inc., Beijing, China) for 24 h. Cells in the loganin treatment group were treated with 100 μM loganin (Macklin, Shanghai, China) for 1 h before TGF‐β1 treatment. Besides, to confirm whether EGFR mediates the regulation of loganin in liver injury, cells were treated with 50 ng/mL EGF (MedChemExpress, Shanghai, China) for 24 h or treated with 5 μM AG‐1478 (A860953, Macklin, China) for 2 h.

### 2.6. Quantitative Real‐Time PCR (qPCR) Analysis

Total RNA was isolated from liver tissues and HSC‐6T cells using the TRIpure reagent (BioTeke, Beijing, China). The RNA samples obtained were reverse‐transcribed into cDNA using the BeyoRT II M‐MLV reverse transcriptase (Beyotime, Shanghai, China) and RNase inhibitor (Biosharp, Hefei, China). The relative mRNA levels of genes were quantified using SYBR green (Solarbio, Beijing, China) and 2 × Taq PCR MasterMix (Solarbio, Beijing, China). The primers used were listed as follows: collagen type I alpha 1 chain (COL1α1) forward: 5′‐CTCAGCCCTCTGTGCCT‐3′, reverse: 5′‐CCTTCGCTTCCATACTCG‐3′; α‐SMA forward: 5′‐TGACCCTGAAGTATCCG‐3′, reverse: 5′‐TCCAGAGTCCAGCACAA‐3′; and fibronectin forward: 5′‐GGATCCCCTCCCAGAGAAGT‐3′, reverse: 5′‐GGGTGTGGAAGGGTAACCAG‐3′. The quantification was normalized to β‐actin and calculated using the formula 2^−ΔΔCt^.

### 2.7. Western Blot

Proteins were extracted using cell lysis buffer (Beyotime, Shanghai, China) containing 1 mM PMSF. After the determination of protein concentration, proteins were separated by SDS‐PAGE and then transferred to polyvinylidene fluoride (PVDF) membranes (Millipore, USA). Subsequently, the membranes were blocked for 1 h, followed by the incubation of primary antibodies at 4°C overnight, including anti‐EGFR (1:1000, AF6043, Affinity), anti‐p‐EGFR (1:1000, AF3045, Affinity), anti‐Beclin‐1 (1:1000, A21191, ABclonal), anti‐LC3B‐II/I (1:1000, A19665, ABclonal), and anti‐β‐actin (1:1000, sc‐47778, Santa Cruz, CA, USA). Afterwards, these membranes were incubated with the secondary antibodies, including HRP‐labeled goat antimouse IgG (1:5000, A0216, Beyotime) and goat antirabbit IgG (1:5000, A0208, Beyotime) for 1 h at room temperature. The blots were visualized by enhanced chemiluminescent (ECL) reagents (Beyotime, Shanghai, China).

### 2.8. MTT Assay

Cells (5 × 10^3^ per well) were seeded in 96‐well plates. Cell viability was assessed in different treatments. Cells treated with different concentrations of loganin (0, 6.25, 12.5, 25, 50, 100, and 200 μM) for 24 h were followed by the MTT examination. Besides, cells were treated with loganin (0, 12.5, 25, 50, and 100 μM) for 1 h separately and then incubated with 10 ng/mL TGF‐β1 for 24 h. Additionally, cells treated with 100 μM loganin for 1 h were cultured in complete medium containing 10 ng/mL TGF‐β1 for 24 h. Subsequently, these cells were incubated in the incubator for 4 h after the addition of 50 μL MTT staining reagent (Biosharp, Hefei, China) per well. Cells were covered with 150 μL DMSO following the removal of the supernatant, and then the optical density (OD) value at 490 nm was detected by a microplate reader (BioTek, Winooski, VT, USA).

### 2.9. Double Immunofluorescence Staining

Liver tissues were embedded in paraffin and cut into slices with a thickness of 5 μm. After blocking in 1% BSA (Sangon Biotech, Shanghai, China) for 15 min, the sections were incubated with anti‐α‐SMA (1:1000, 14395‐1‐AP, Proteintech) and anti‐EGFR (1:200, AF6043, Affinity) or anti‐α‐SMA and anti‐LC3B (1:50, sc‐271625, Santa Cruz) at 4°C overnight at the same time. Following the incubation with secondary antibodies (FITC‐labeled antirabbit IgG [1:200, #4413, CST] and Cy3‐labeled antimouse IgG [1:200, #4408, CST]) for 90 min in the dark, the sections were stained with DAPI (Aladdin, Shanghai, China) to redye the nucleus. The fluorescence images were observed and photographed using a BX53 microscope (400×) (Olympus, Tokyo, Japan).

### 2.10. Transmission Electron Microscopy (TEM)

Cells were fixed with the specific stationary liquid at 4°C. After fixation, dehydration, and embedding, the cells were made into ultrathin sections and stained. The intracellular changes were observed under TEM, and the images were collected.

### 2.11. Evaluation of Autophagy

HSC‐T6 cells were infected with mRFP‐GFP‐LC3 double fluorescent lentivirus for 24 h. Cells were treated with 100 μM loganin for 1 h and then treated with 10 ng/mL TGF‐β1 for 24 h. Then, cells were collected and observed using a fluorescence microscope (400×).

### 2.12. Statistical Analysis

All statistical analyses were carried out using GraphPad Prism 8.0. Data were presented from six independent experiments involving animals (*N* = 6) and three independent experiments involving cells (*N* = 3) to ensure the accuracy and completeness of the experimental results. Data were presented as mean ± SD. For comparison between the two groups, an unpaired *t*‐test was employed. For comparisons among multiple groups, one‐way ANOVA followed by Tukey’s multiple comparisons test or Dunnett’s multiple comparisons test was applied. Statistical significance was set at *p* < 0.05.

## 3. Results

### 3.1. Loganin Alleviates Liver Injury and Hepatic Fibrosis in BDL Rats

The 2D structure of loganin is shown in Figure 1A. To establish the liver injury and hepatic fibrosis model, Sprague–Dawley rats underwent BDL surgery following 1 week of acclimatization (Figure 1B). After 24 h of postoperative recovery, rats received daily intraperitoneal injections of loganin (7 mg/kg) for 14 consecutive days, after which tissue and serum samples were collected for further analysis (Figure 1C).

Figure 1Loganin ameliorates liver injury and hepatic fibrosis in a bile duct ligation (BDL) rat model. (A) Chemical structure of loganin. (B) Schematic diagram of the BDL in Sprague–Dawley rats. (C) Paradigm of BDL model establishment and loganin treatment. (D) Photographs of the liver (scale bar: 1 cm) and hematoxylin and eosin (H&E) staining (scale bar: 100 μm) of liver tissues. (E) The ratio of liver weight to body weight of rats was calculated (*N* = 6). (F) The levels of alanine aminotransferase (ALT), aspartate aminotransferase (AST), and total bilirubin (T‐BIL) in serum were determined (*N* = 6). (G) Images of liver sections stained with Masson and Sirius Red. Scale bars: 100 μm. (H) Hydroxyproline (HYP) content was assessed in liver tissue using a detection kit (*N* = 6). (I) The mRNA levels of collagen type I alpha 1 chain (COL1α1), alpha‐smooth muscle actin (α‐SMA), and fibronectin in liver tissue were estimated using qPCR (*N* = 6). Mean ± SD,  ^∗^ *p* < 0.05,  ^∗∗^ *p* < 0.01.

**Figure:**
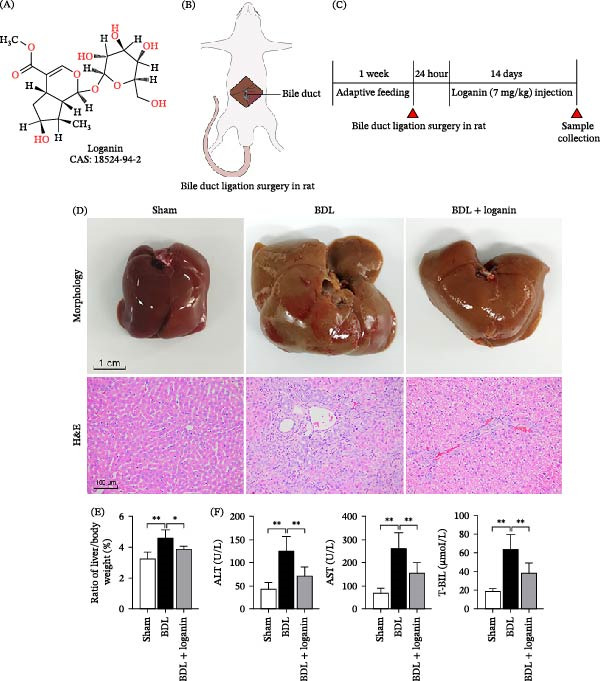

**Figure:**
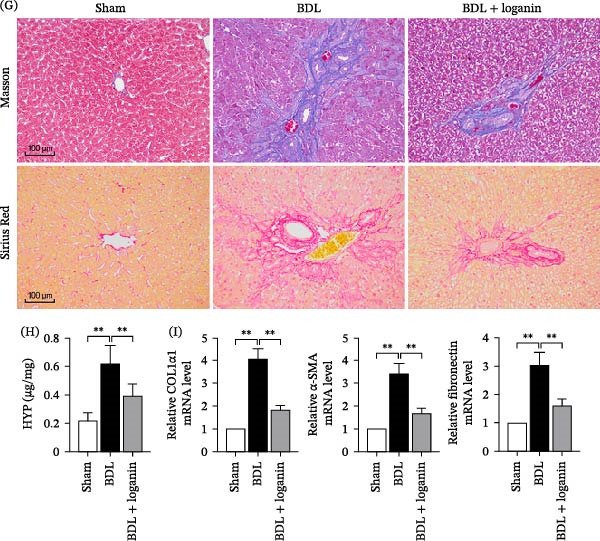

To exclude the basal effects of loganin on normal liver function, a sham + loganin group was added. Results showed that loganin did not significantly affect liver tissue damage (Figure S1A), collagen deposition (Figure S1B), liver/body weight ratio (Figure S1C), serum ALT/AST/bilirubin (Figure S1D), or hepatic HYP content (Figure S1E) versus the sham group. These data confirm that loganin alone does not induce liver injury in healthy rats. After that, we evaluated the effect of loganin on the liver function of BDL rats. BDL surgery induced marked hepatomegaly, tissue adhesion, and surface irregularity of the liver, all of which were substantially ameliorated by loganin treatment (upper panel, Figure 1D). Histological analysis by H&E staining further revealed severe inflammatory infiltration and hepatocyte necrosis in BDL rats, whereas loganin administration significantly attenuated these pathological changes (lower panel, Figure 1D). Consistently, the elevated liver‐to‐body weight ratio observed in BDL rats was significantly reduced following loganin intervention (Figure 1E). Serum biochemical assays demonstrated that BDL markedly elevated the levels of ALT, AST, and T‐BIL, indicating severe hepatocellular damage, while loganin treatment effectively restored these parameters (Figure 1F). Furthermore, Sirius Red staining revealed prominent collagen fiber deposition in BDL liver tissues, which was significantly diminished by loganin administration (Figure 1G). This antifibrotic effect was corroborated by HYP quantification, showing that loganin markedly suppressed collagen overproduction (Figure 1H). At the molecular level, BDL‐induced upregulation of fibrogenic genes, including COL1α1, α‐SMA, and fibronectin, was significantly attenuated by loganin treatment (Figure 1I). Collectively, these findings demonstrated that loganin exerts a protective effect against BDL‐induced cholestatic liver injury and hepatic fibrosis in rats.

### 3.2. Loganin Attenuates Hepatic Autophagy in BDL Rats

**Figure 2:**
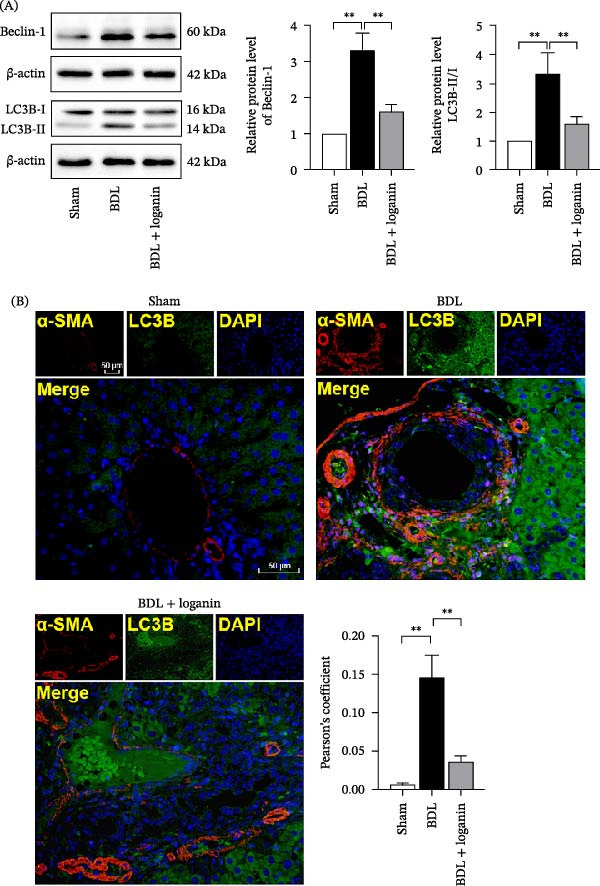
Loganin suppresses autophagy in the liver tissues of BDL rats. (A) Protein expression levels of Beclin‐1 and the LC3B‐II/I ratio were assessed by Western blot analysis, and quantitative data were obtained (*N* = 6). (B) Immunofluorescence double staining was performed to detect LC3B and α‐SMA expression in liver tissue, and colocalization was quantified using Pearson’s correlation coefficient (*N* = 6). Scale bars: 50 μm. Mean ± SD,  ^∗∗^ *p* < 0.01.

Emerging evidence indicates that loganin possesses autophagy‐inhibitory properties [12], while autophagy has been implicated as a key regulator of HSC activation and liver fibrosis progression [20]. Accordingly, we examined the effects of loganin on autophagic activity in the livers of BDL rats. Autophagy initiation is mediated by Beclin‐1, which promotes autophagosome formation [21]. During autophagosome assembly, LC3B‐I is recruited and converted to LC3B‐II, the latter serving as a hallmark of autophagy activation [22]. As shown in Figure 2A, BDL treatment significantly upregulated Beclin‐1 expression and increased the LC3B‐II/I ratio in liver tissue, both of which were markedly suppressed by loganin, indicating that loganin attenuates hepatic autophagy. Furthermore, double immunofluorescence staining revealed that the expressions of α‐SMA and LC3B were both prominently elevated in BDL rats. In contrast, loganin treatment effectively reduced their colocalization (Figure 2B). Collectively, these findings suggested that loganin alleviates liver injury and hepatic fibrosis, at least in part, through the suppression of autophagy.

### 3.3. Screening of Loganin Downstream Factors and Liver Fibrosis–Related Proteins

To systematically identify the potential targets of loganin involved in liver fibrosis regulation, we performed a series of bioinformatics analyses to determine the key targets. A total of 100 targets of loganin were identified using the SwissTargetPrediction online tool (Figure 3A). Meanwhile, liver fibrosis–related proteins were downloaded from the GeneCards database, and Venny analysis was used to obtain 78 intersection factors between loganin targets and liver fibrosis‐related targets (Figure 3B), which were considered as potential targets of loganin in regulating liver fibrosis. To further screen the core target from these 78 intersection factors, the overlap targets were submitted to the STRING database for PPI network construction, and protein interaction data with a confidence score ≥ 0.4 were visualized (Figure 3C). Among all nodes in the PPI network, EGFR showed the highest interaction score, suggesting that EGFR might be a key hub protein in the regulatory network of loganin against liver fibrosis.

Figure 3Screening of loganin downstream factors. (A) Classification of the target proteins of loganin predicted by SwissTargetPrediction. (B) Venn diagram of loganin target proteins and hepatic fibrosis–related proteins. (C) Construction of the PPI network at the overlap proteins of loganin targets and hepatic fibrosis–related proteins. (D) The top 10 GO enrichment analyses for overlap targets were listed. (E) KEGG pathway secondary enrichment analysis of overlap targets.

**Figure:**
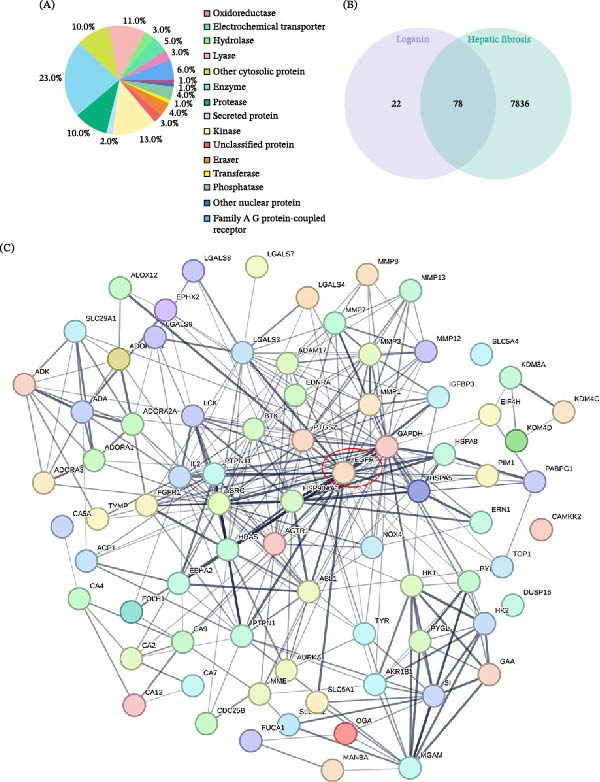

**Figure:**
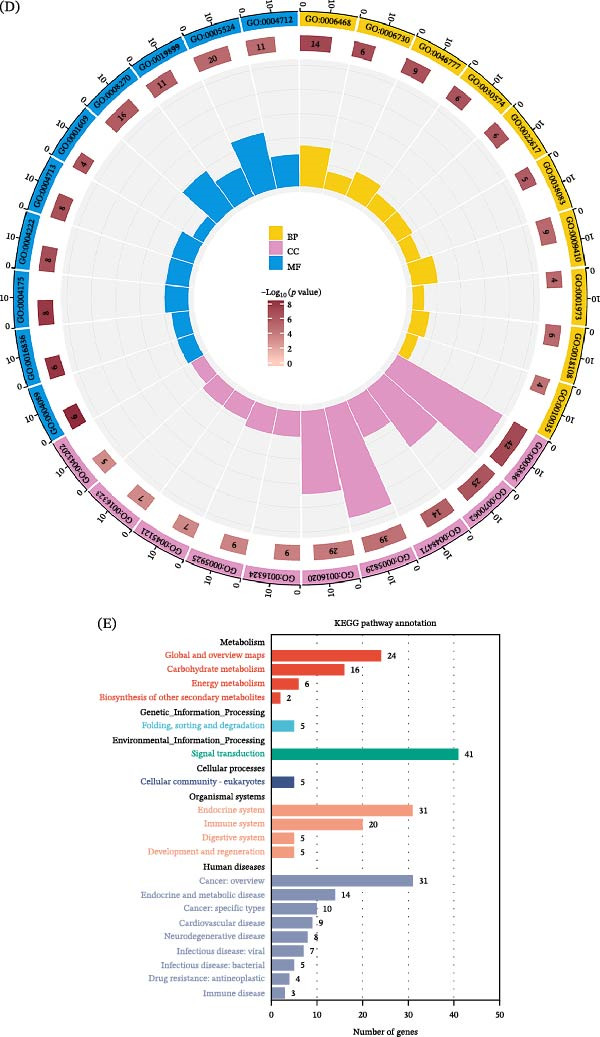

To verify this hypothesis and explore the functional relevance of EGFR, we analyzed the GO and KEGG enrichment results of the 78 intersection targets. The top 10 enriched terms of biological process (BP), cellular component (CC), and molecular function (MF) categories in GO enrichment results were visualized (Figure 3D). In the BP category, the targets were significantly enriched in protein phosphorylation (GO:0006468, *p*  < 0.01, FDR < 0.05), a process consistent with the known mechanism of EGFR signaling activation via autophosphorylation. In the CC category, the overlap proteins were predominantly localized to the plasma membrane (GO:0005886, *p*  < 0.01), aligning with the subcellular distribution of membrane‐bound receptors, including EGFR. In the MF category, ATP binding (GO:0005524) was the most significantly enriched term (*p* < 0.001), compatible with the kinase activity characteristic of EGFR and other ATP‐dependent kinases present in the target set. The KEGG enrichment analysis identified a total of 60 pathways, and we then conducted KEGG secondary classification for the significantly enriched pathways (*p* < 0.05) and visualized the results (Figure 3E). Notably, the signal transduction pathway was the most significantly enriched term. According to the reported study, the EGFR signaling pathway has been implicated in liver fibrosis progression [23]. Collectively, these bioinformatics findings suggested that the EGFR signaling pathway may represent a candidate target contributing to the antifibrotic effects of loganin. However, further experimental validation is required to establish the causal relationship between EGFR and loganin.

### 3.4. Loganin Inhibits the Activation of the EGFR Signaling Pathway in BDL Rats

Molecular docking analysis revealed that loganin forms hydrogen bonds with EGFR, suggesting that there might be a potential interaction between loganin and this receptor (Figure 4A). To experimentally validate whether loganin ameliorates BDL‐induced liver injury and hepatic fibrosis via the EGFR signaling pathway, EGFR protein expression was assessed in liver tissue. As shown in Figure 4B, BDL‐induced upregulation of p‐EGFR was remarkably suppressed by loganin treatment. Furthermore, immunofluorescence staining demonstrated that EGFR signaling was activated alongside HSC activation in BDL rats, as evidenced by the increased expression of EGFR and α‐SMA; these effects were significantly attenuated by loganin (Figure 4C). Collectively, these findings suggested that EGFR signaling partially mediates the hepatoprotective effects of loganin against BDL‐induced injury and fibrosis.

Figure 4Effect of loganin on the activation of EGFR in the liver tissue of the BDL rats. (A) The binding interaction between loganin and EGFR was predicted by molecular docking analysis. (B) The protein expression of EGFR and phosphorylated EGFR in liver tissues was assessed by Western blot, and quantitative data were obtained (*N* = 6). (C) Immunofluorescence double staining was used to evaluate the expression of EGFR and α‐SMA in liver tissue, and colocalization was quantified using Pearson’s correlation coefficient (*N* = 6). Scale bars: 50 μm. Mean ± SD,  ^∗∗^ *p* < 0.01.

**Figure:**
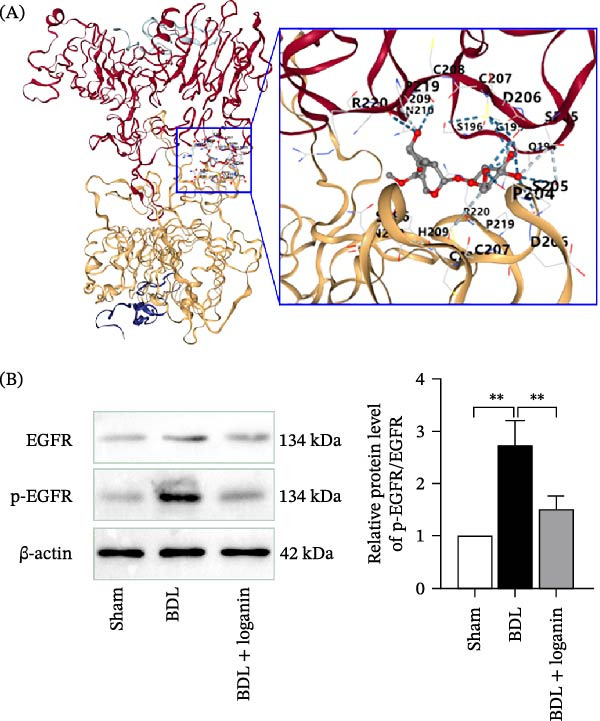

**Figure:**
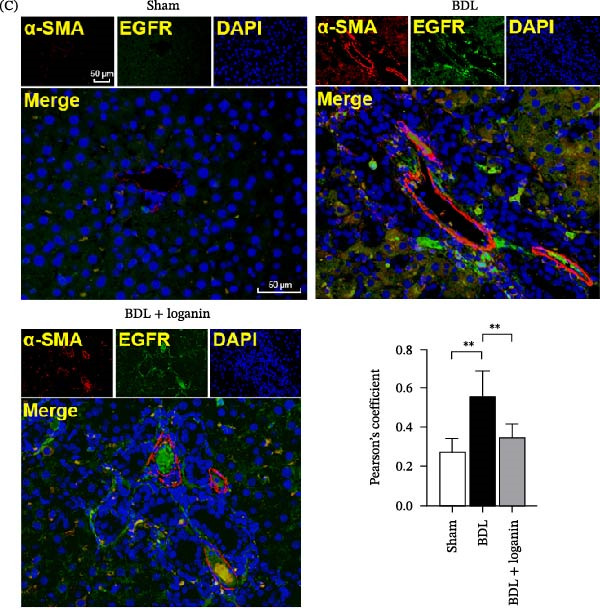

### 3.5. Loganin Inhibits the Activation and Autophagy Induced by TGF‐β1 in HSC‐T6 Cells

HSC activation represents the central event in hepatic fibrogenesis [11]. In order to verify the effect of loganin in an in vitro environment, HSC‐T6 cells were treated with TGF‐β1 (10 ng/mL) for 24 h to establish an activation model. To determine the optimal concentration of loganin, cell viability was assessed by the MTT assay. As shown in Figure 5A, loganin at 200 μM significantly suppressed basal cell viability compared with the blank group (*p*  < 0.01), indicating a potential cytotoxicity at higher concentrations. In TGF‐β1–activated cells, loganin at 50 μM and 100 μM significantly inhibited cell viability in a concentration‐dependent manner (*p*  < 0.05 and *p*  < 0.01, respectively, Figure 5B). Notably, 100 μM loganin exhibited a more pronounced effect without reaching the cytotoxic threshold observed at 200 μM. Based on these results, 100 μM loganin was selected for subsequent experiments.

Figure 5Effects of loganin on the activation and autophagy of TGF‐β1‐stimulated HSC‐T6 cells. (A) HSC‐T6 cells were treated with different concentrations of loganin, and the MTT assay was used to detect cell viability (*N* = 3). (B) TGF‐β1–stimulated HSC‐T6 cells were treated with different concentrations of loganin, and cell viability was measured (*N* = 3). (C) The effect of loganin on cell viability of TGF‐β1–stimulated HSC‐T6 cells (*N* = 3). (D) The mRNA levels of COL1α1, α‐SMA, and fibronectin in TGF‐β1–stimulated HSC‐T6 cells (*N* = 3). (E) Transmission electron microscopy was used to observe and inspect autophagy in HSC‐T6 cells. Blue arrows pointed to autolysosomes, and red arrows pointed to autophagosomes. Scale bar = 1 μm. (F, G) Protein expression levels of Beclin‐1 and the LC3B‐II/I ratio in TGF‐β1–stimulated HSC‐T6 cells were assessed, and quantitative data were obtained (*N* = 3). (H, I) HSC‐T6 cells were infected with mRFP‐GFP‐LC3 double fluorescent lentivirus, and the proportion of autophagosomes (yellow dots) and autolysosomes (red dots) was quantified (*N* = 3). Mean ± SD,  ^∗^ *p* < 0.05,  ^∗∗^ *p* < 0.01.

**Figure:**
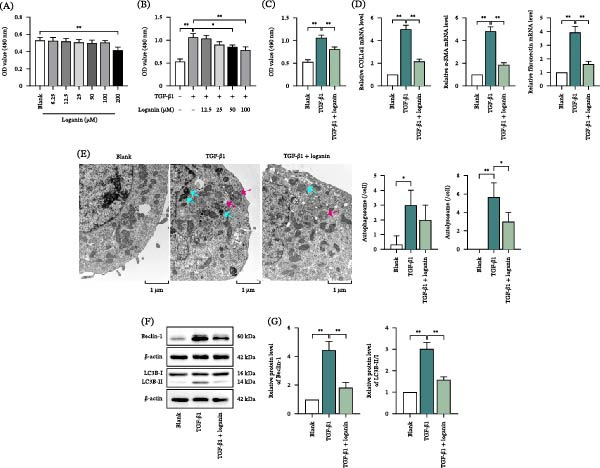

**Figure:**
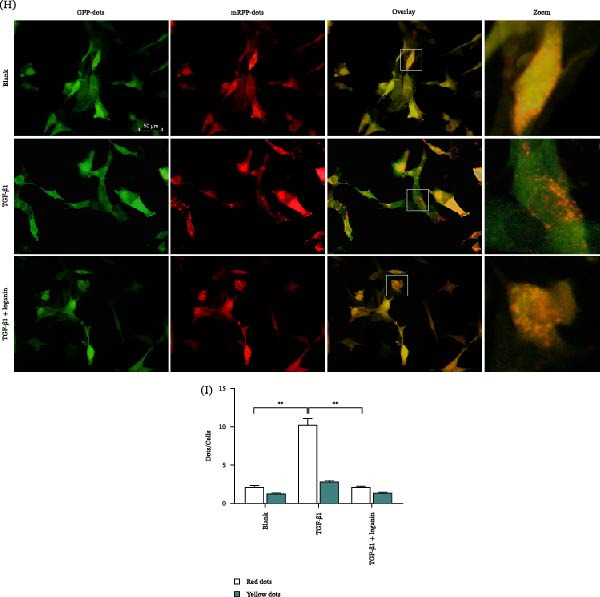

To exclude the basal effects of loganin on HSC‐T6 cells, a blank + loganin group was added (Figure S2). Results showed that loganin alone (without TGF‐β1) did not significantly alter fibrogenic markers (Figure S2A) or autophagy‐related proteins (Figure S2B) versus the blank group. These data confirm that loganin does not induce HSC activation or autophagy in unstimulated cells. However, loganin effectively suppressed TGF‐β1–induced HSC‐T6 cell activation (Figure 5C) and significantly downregulated the mRNA expression of fibrosis markers, including COL1α1, α‐SMA, and fibronectin, that were upregulated by TGF‐β1 (Figure 5D). Collectively, these findings demonstrated that loganin alleviates TGF‐β1–induced activation in HSC‐T6 cells.

We next examined the effect of loganin on autophagy in TGF‐β1–stimulated HSC‐T6 cells. TEM analysis revealed that TGF‐β1 significantly increased autophagosomes and autolysosomes compared to the blank group, whereas loganin markedly reduced the autolysosome level (Figure 5E), suggesting impaired autophagic flux. This morphological observation was corroborated by Western blot analysis, showing that loganin restored TGF‐β1–upregulated expression of Beclin‐1 and LC3B‐II/I (Figure 5F,G). To further elucidate autophagic dynamics, the GFP‐RFP‐LC3 dual‐fluorescence assay showed that TGF‐β1 significantly increased red puncta with minimal change in yellow puncta, resulting in an enhanced autophagic flux; conversely, loganin inhibited this trend compared to the TGF‐β1 group (Figure 5H,I). Collectively, these data demonstrated that loganin may suppress TGF‐β1–induced HSC activation by inhibiting autophagic flux.

### 3.6. EGFR Signaling Mediates the Antifibrotic Effect of Loganin in TGF‐β1–Sitimulated HSCs

**Figure 6:**
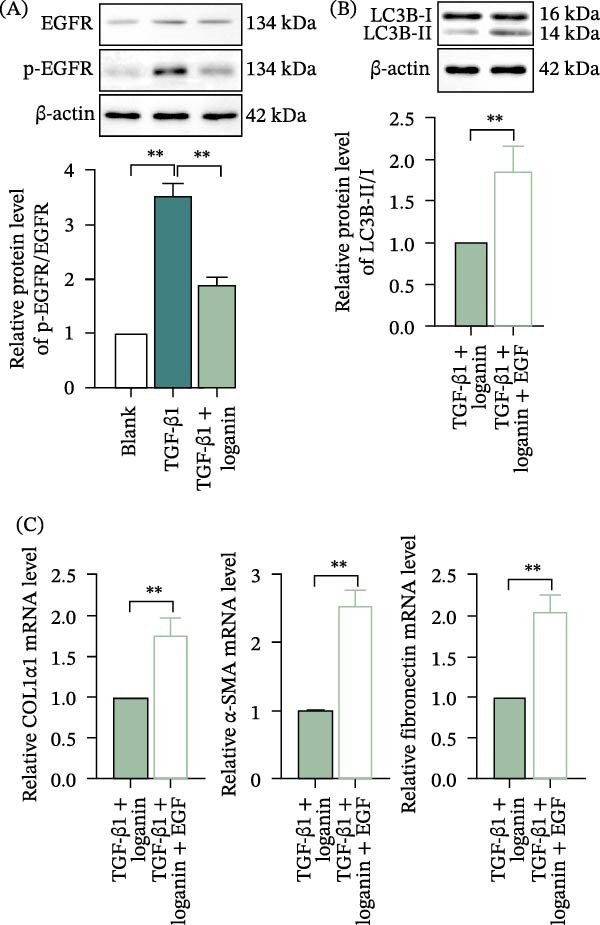
Loganin inhibits the activation and autophagy of HSC‐T6 cells by blocking EGFR signaling. (A) The protein expression of EGFR and phosphorylated EGFR in HSC‐T6 cells was assessed by Western blot, and quantitative data were obtained (*N* = 3). (B) The protein expression of LC3B‐II/I in HSC‐T6 cells was assessed by Western blot, and quantitative data were obtained (*N* = 3). (C) The effect of EGF on mRNA levels of COL1α1, α‐SMA, and fibronectin in TGF‐β1–stimulated HSC‐T6 cells (*N* = 3). Mean ± SD,  ^∗∗^ *p* < 0.01.

Based on the above results, we examined the effect of EGFR phosphorylation in TGF‐β1–sitimulated HSC‐T6 cells. Loganin significantly suppressed TGF‐β1–induced p‐EGFR upregulation (Figure 6A), indicating the inhibition of EGFR pathway activation. To functionally validate this mechanism, rescue experiments were performed using EGF (an EGFR agonist). Compared with loganin treatment alone, EGF cotreatment significantly restored LC3B‐II/I expression (Figure 6B), demonstrating that EGFR activation counteracts the autophagy‐inhibitory effect of loganin. Consistently, EGF also reversed loganin‐induced downregulation of fibrogenic markers (COL1α1, α‐SMA, and fibronectin) (Figure 6C). To further corroborate these findings, the pharmacological EGFR inhibitor AG‐1478 was employed as a positive control. AG‐1478 significantly suppressed TGF‐β1–induced fibrotic markers (Figure S3A) and decreased the LC3B‐II/I ratio (Figure S3B), mirroring the effects of loganin. Collectively, these results demonstrated that loganin attenuates hepatic fibrosis, at least in part, through the suppression of EGFR signaling.

## 4. Discussion

**Figure 7:**
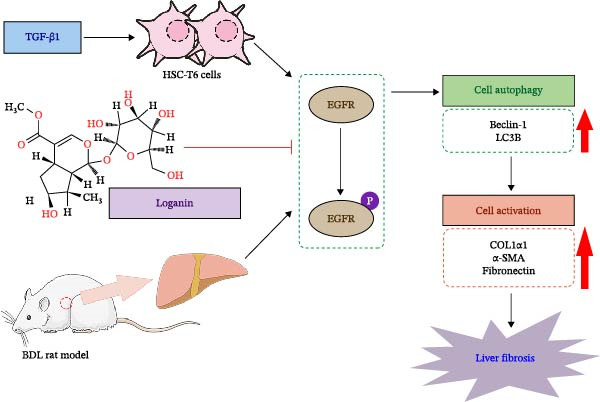
Loganin ameliorates liver fibrosis by inhibiting autophagy and activation of HSCs through regulating the EGFR signaling pathway.

In this study, we elucidated the antifibrotic mechanism of loganin through integrated network pharmacology prediction and experimental validation. We demonstrated that loganin significantly ameliorated liver injury and hepatic fibrosis in BDL rats and suppressed TGF‐β1–induced HSC activation and autophagy. Mechanistically, these effects were mediated through the blockade of EGFR signaling (Figure 7). Collectively, our findings indicated that loganin attenuates liver injury and hepatic fibrosis by inhibiting HSC activation and autophagy via the modulation of the EGFR pathway.

Loganin has demonstrated therapeutic potential in various pathological conditions. Notably, it was identified as an effective agent for mitigating Parkinson’s disease through suppression of autophagy, apoptosis, and inflammation [24]. Additionally, loganin ameliorated inflammatory responses in nonalcoholic steatohepatitis by attenuating inflammasome expression in the liver [10], highlighting its broad regulatory capacity for hepatic inflammation. The liver, a vital organ with complex metabolic functions, is particularly vulnerable to inflammatory and fibrotic insults that can precipitate organ dysfunction and irreversible damage [25]. Cholestatic liver injury, commonly resulting from bile duct obstruction, represents a significant clinical challenge frequently progressing to hepatic fibrosis [26]. The activation and proliferation of HSCs constitute the central pathogenic mechanism driving this fibrotic process [27]. Following BDL, accumulated bile acids induce hepatocellular damage, triggering HSC activation and subsequent ECM deposition. Importantly, advanced fibrosis becomes increasingly refractory to treatment and carries a substantial risk for cirrhosis development [28]. Therefore, we employed the BDL model to establish cholestatic liver injury and hepatic fibrosis in rats. Consistent with previous reports [29, 30], BDL surgery induced marked hepatomegaly, elevated serum transaminases and bilirubin, and prominent collagen deposition. Notably, loganin treatment significantly ameliorated these pathological changes without compromising normal liver physiology, as evidenced by the absence of adverse effects in sham‐operated rats. The observed reduction in the liver‐to‐body weight ratio and restoration of serum biochemical parameters further corroborate the hepatoprotective efficacy of loganin against cholestatic injury.

Autophagy functions as a lysosomal degradation pathway that recycles intracellular components to generate metabolic energy and biosynthetic precursors, thereby sustaining cellular homeostasis and renewal [31]. Previous reports have indicated that the obstruction of autophagy led to the suppression of HSC activation, which is a potential therapeutic strategy for liver injury and hepatic fibrosis [32, 33]. Inhibition of HSC activation and autophagy is an important and effective method to inhibit liver fibrosis [34]. Besides, loganin has been reported to prevent neuronal autophagy through reducing the expression of Beclin‐1 and LC3B‐II/I [15]. Our findings revealed that BDL surgery substantially upregulated hepatic autophagy markers, including Beclin‐1 and LC3B‐II/I ratio, concomitant with HSC activation. Importantly, loganin administration markedly suppressed these autophagic indicators and reduced colocalization of α‐SMA with LC3B, suggesting that autophagy inhibition contributes to its antifibrotic mechanism. These results align with previous studies demonstrating that genetic or pharmacological inhibition of autophagy attenuates HSC activation and fibrosis progression [13, 14].

To elucidate the molecular target underlying loganin, we conducted comprehensive bioinformatics analyses integrating drug target prediction, disease‐associated proteins, and pathway enrichment. Among 78 intersecting targets, EGFR emerged as the top hub protein in the PPI network, with significant enrichment in protein phosphorylation and signal transduction pathways. EGFR, a receptor tyrosine kinase traditionally associated with cell proliferation and cancer, has gained recognition as a critical regulator of HSC activation and fibrogenesis [35]. Upon ligand binding, EGFR undergoes autophosphorylation and initiates downstream cascades, which promote HSC survival, migration, and ECM production [36]. Our molecular docking analysis supported a predicted relationship between loganin and EGFR, which was functionally validated by the suppression of EGFR phosphorylation in BDL rat livers and TGF‐β1–activated HSC‐T6 cells. These findings positioned EGFR as an important target mediating the antifibrotic effects of loganin.

Our in vitro studies in HSC‐T6 cells provided additional mechanistic insights into the autophagy‐modulatory effects of loganin. TGF‐β1, a potent profibrogenic cytokine, induced significant HSC activation and autophagy [37]. Notably, loganin treatment markedly reduced autolysosome formation, which is consistent with the effect of loganin on autophagy in chronic constriction injury [15]. Finally, the functional significance of EGFR targeting was further substantiated through rescue experiments using EGF and the pharmacological EGFR inhibitor AG‐1478.

In addition, this study also has several limitations that should be acknowledged. First, our findings are derived exclusively from preclinical models (rodent BDL model and HSC‐T6 cell line) without clinical data from liver disease patients; therefore, the efficacy and safety of loganin in human subjects remain to be established. Second, our in vitro experiments were performed only under TGF‐β1 stimulation, whereas liver fibrosis is driven by multiple profibrotic factors, like PDGF‐BB; whether loganin exerts similar inhibitory effects against other fibrogenic stimuli in liver fibrosis requires further investigation. Third, although molecular docking predicted a potential interaction between loganin and EGFR, rigorous validation of direct binding through biophysical assays was not performed due to technical limitations; thus, we cannot conclusively demonstrate direct molecular interaction, and our data support that loganin exerts antifibrotic effects by inhibiting EGFR signaling without claiming a direct mechanism. Fourth, loganin has been traditionally used for anti‐inflammation, antidiabetes, and hepatoprotection but not specifically for wound healing or tissue repair; given that liver fibrosis involves complex wound healing responses, the effects of loganin on hepatic injury repair in clinical settings require careful evaluation. Future studies are warranted to address these limitations and further elucidate the therapeutic potential of loganin.

In conclusion, this study demonstrated that loganin alleviates cholestatic liver injury and hepatic fibrosis through inhibition of EGFR signaling and subsequent suppression of autophagy and activation of HSCs. These findings may provide mechanistic insights into the antifibrotic properties of loganin.

## Author Contributions

**Xinwen Li, Huiling Zhang, and Chao Zhang**: conceptualization, data curation, investigation, validation, writing – original draft, writing – review and editing. **Xiaomin Zhang**: data curation, investigation, validation, visualization. **Jiefeng He**: conceptualization, methodology, project administration, writing – review and editing.

## Funding

The authors have nothing to report.

## Ethics Statement

The animal experimental procedures reported in this article were approved by the Ethics Committee of Shanxi Bethune Hospital (Approval Number YXLL‐2022‐006).

## Conflicts of Interest

The authors declare no conflicts of interest.

## Supporting Information

Additional supporting information can be found online in the Supporting Information section.

## Supporting information


**Supporting Information** Figure S1: The effect of loganin on the liver function of the sham rat. (A, B) H&E and Sirius Red staining images of liver tissues from sham and sham + loganin rats. (C) The ratio of liver weight to body weight of rats was calculated (*N* = 6). (D) The levels of ALT, AST, and T‐BIL in serum were determined (*N* = 6). (E) HYP content was assessed in liver tissue using a detection kit (*N* = 6). Mean ± SD,  ^∗^ *p* < 0.05,  ^∗∗^ *p* < 0.01. Figure S2: The effect of loganin on HSC‐T6 cell activation without TGF‐β1 stimulation. (A) The mRNA levels of COL1α1, α‐SMA, and fibronectin in loganin‐treated HSC‐T6 cells (*N* = 3). (B) Protein expression levels of Beclin‐1 and the LC3B‐II/I ratio in loganin‐treated HSC‐T6 cells were assessed, and quantitative data were obtained (*N* = 3). Mean ± SD,  ^∗^ *p* < 0.05,  ^∗∗^ *p* < 0.01. Figure S3: The effect of loganin and AG‐1478 on TGF‐β1–stimulated HSC‐T6 cells. (A) The effect of loganin and AG‐1478 on mRNA levels of COL1α1, α‐SMA, and fibronectin in TGF‐β1–treated HSC‐T6 cells (*N* = 3). (B) The effect of loganin and AG‐1478 on the protein expression level of LC3B‐II/I in TGF‐β1–treated HSC‐T6 cells was assessed, and quantitative data were obtained (*N* = 3). Mean ± SD,  ^∗^ *p* < 0.05,  ^∗∗^ *p* < 0.01.

## Data Availability

The data that support the findings of this study are available from the corresponding author upon reasonable request.
