# Supplementary material for: Loganin Ameliorates Cholestatic Liver Fibrosis by Inhibiting Hepatic Stellate Cell Autophagy and Activation Through EGFR Signaling
Source: Mediators Inflamm. 2026 Aug 17;2026:7967581. doi: 10.1155/mi/7967581 (PMC13478915; doi:10.1155/mi/7967581)
Supplement: Supplementary file 1 [file MI-2026-7967581-s001.docx]

Supplementary Figures：

Figure S1 The effect of Loganin on the liver function of the sham rat

A-B. H&E and Sirius Red staining images of liver tissues from sham and sham + loganin rats. C. The ratio of liver weight to body weight of rats was calculated (N = 6). D. The levels of ALT, AST, and T-BIL in serum were determined (N = 6). E. HYP content was assessed in liver tissue using a detection kit (N = 6). Mean ± SD, *P < 0.05, **P < 0.01.

Figure S2 The effect of Loganin on HSC-T6 cell activation without TGF-β1 stimulation

A. The mRNA levels of COL1α1, α-SMA, and fibronectin in Loganin-treated HSC-T6 cells (N = 3). B. Protein expression levels of Beclin-1 and the LC3B-II/I ratio in Loganin-treated HSC-T6 cells were assessed, and quantitative data were obtained (N = 3). Mean ± SD, *P < 0.05, **P < 0.01.

Figure S3 The effect of Loganin and AG-1478 on TGF-β1 stimulated HSC-T6 cells

A. The effect of Loganin and AG-1478 on mRNA levels of COL1α1, α-SMA, and fibronectin in TGF-β1-treated HSC-T6 cells (N = 3). B. The effect of Loganin and AG-1478 on the protein expression level of LC3B-II/I in TGF-β1-treated HSC-T6 cells was assessed, and quantitative data were obtained (N = 3). Mean ± SD, *P < 0.05, **P < 0.01.
